# Ecological Momentary Assessments: A Real-World Solution for Understanding Functional Challenges

**DOI:** 10.1192/j.eurpsy.2025.1448

**Published:** 2025-08-26

**Authors:** S. Volovic Shushan, A. Stern, N. Josman

**Affiliations:** 1 The Faculty of Social Welfare and Health Sciences, Department of Occupational Therapy, University of Haifa, Haifa; 2 ”Clalit” health services, “Shalvata” Mental Health Hospital, Hod-Ha’Sharon; 3 Faculty of Health Sciences, Occupational Therapy Department, Ben-Gurion University of the Negev, Beer Sheva, Israel

## Abstract

**Introduction:**

Major Depressive Disorder (MDD) is the leading cause of disability worldwide, affecting individuals’ functioning in various life areas. Prolonged residual functional impairment is one of the risk factors for recurrence. Moreover, symptoms severity and accompanying functional disabilities negatively impact quality of life (QoL) and personal well-being, affecting the recovery process of people with MDD and their reintegration into daily life. Therefore, restoring functional abilities is no less important than reducing symptoms.

**Objectives:**

Recent changes in mental health policy have led to an expansion of client-oriented community-based services, focusing on preventing health problems and promoting QoL and well-being. A significant change can also be seen in depression evaluation and treatment, moving from traditional face-to-face therapy to hybrid care settings that incorporate remote or home-based treatments and assessments of everyday life.

While traditional assessments of symptoms and behavior often rely on questionnaires and interviews, they frequently miss the dynamic changes in daily functioning experienced by people with MDD. Clinicians primarily rely on patients’ retrospective reports regarding mood, affective state, thoughts, and behavior. However, understanding and gaining insight into day-to-day experiences requires addressing dynamic processes and changes that occur over time, rather than in a single time point. Hence, **ecological momentary assessment** (EMA) is a powerful and effective technique for assessing moment-to-moment function patterns in daily life.

**Methods:**

Advancements in technology have enabled the use of computer-assisted methodology and real-time monitoring EMAs. The methodological advantages, including the circumvention of retrospective bias and increased longitudinal and ecological validity, have facilitated the widespread use of EMA in mood disorder clinical practice. Nevertheless, addressing everyday functioning using EMA remains limited in clinical research and practice.

**Results:**

Utilizing EMA can enhance our understanding of human experience, leading to human-centered research, design practice, and mental health care. It has the potential to reveal real everyday functioning and reflect the activities and contexts chosen and experienced by people with MDD. Addressing each patient’s unique functional profile can facilitate personalized interventions, supporting the recovery process and improving QoL.

**Conclusions:**

This presentation will review the benefits of EMA in the field of mood traits, and especially EMA monitoring for daily function. Additionally, it will present recent studies using EMA and discuss advancements and clinical applications.

**Disclosure of Interest:**

None Declared

